# Understanding Why Post-Stroke Depression May Be the Norm Rather Than the Exception: The Anatomical and Neuroinflammatory Correlates of Post-Stroke Depression

**DOI:** 10.3390/jcm10081674

**Published:** 2021-04-14

**Authors:** Tissa Wijeratne, Carmela Sales

**Affiliations:** 1 School of Psychology and Public Health, La Trobe University, Melbourne 3000, Australia; 2 Department of Neurology, Western Health & University Melbourne, AIMSS, Level Three, WHCRE, Sunshine Hospital, St Albans 3021, Australia; Carmela.Sales@wh.org.au; 3 Department of Medicine, Faculty of Medicine, University of Rajarata, Saliyapura, Anuradhapura 50000, Sri Lanka

**Keywords:** cytokine, depression, ischemia, stroke, apoptosis, excitotoxicity, oncosis, inflammation

## Abstract

Ischemic Stroke precedes depression. Post-stroke depression (PSD) is a major driver for poor recovery, negative quality of life, poor rehabilitation outcomes and poor functional ability. In this systematic review, we analysed the inflammatory basis of post-stroke depression, which involves bioenergetic failure, deranged iron homeostasis (calcium influx, Na influx, potassium efflux etc), excitotoxicity, acidotoxicity, disruption of the blood brain barrier, cytokine-mediated cytotoxicity, reactive oxygen mediated toxicity, activation of cyclooxygenase pathway and generation of toxic products. This process subsequently results in cell death, maladapted, persistent neuro-inflammation and deranged neuronal networks in mood-related brain regions. Furthermore, an in-depth review likewise reveals that anatomic structures related to post-stroke depression may be localized to complex circuitries involving the cortical and subcortical regions.

## 1. Introduction

Stroke is one of the leading causes of death and disability globally. There are 16 million strokes annually, with 6 million deaths and another 5.5 million left with significant disability with enormous health, social and economic impact worldwide [1]. The direct cost per person over a year post stroke is approximately US$58,200 [2]. Given the high global prevalence and disability of stroke and associated costs for the global community, it is important to explore the factors that may impact on outcome, in order to get the best possible outcomes for the affected patents [1,3,4].

Depression is responsible for heavy global societal burden with more than 258 million people worldwide with rising rates globally [5,6,7,8]. Both stroke and depression have been associated with increased inflammatory activation of the immune system causing negative health impacts in both conditions.

In this review, we explored the relationship between stroke, inflammation and depression, based on systematic review as described below. Furthermore, an extensive review of the anatomical correlates of PSD was also undertaken.

Information about acute ischemic stroke (AIS), depression and inflammation was electronically searched. Only articles in English, involving human subjects and published between 2005 and 2020 were considered (several key pre-2005 publications from first author’s personal collection were also considered).

The following databases were searched with the following keywords: stroke; depression; inflammation and neuroanatomy. The following databases were looked into: MEDLINE; Cochrane and CINAHL. The bibliographies of individual studies were further hand-searched. Articles were screened and evaluated independently by the two investigators. Publications were reviewed in terms of the sample size, design and reliability of data collection.

### 1.1. Poststroke Depression

Depression after stroke is a prevalent yet under recognized complication of stroke. The Secondary Prevention of Small Subcortical Strokes (SPS3) trial revealed that 19% of stroke patients suffered from post-stroke depression (PSD), while a meta-analysis of 51 observational studies estimates that one out of every three stroke survivors suffer from the PSD [9,10]. Patients with PSD have been shown to have poorer neurological outcomes, and its economic costs are staggering, affecting health systems at a global scale [11,12,13,14,15,16,17,18,19,20,21,22,23,24,25].

Prognostic outcome of stroke is dependent on several factors such as age, gender, initial severity of the stroke, functional status at admission to hospital, urinary incontinence, impairment in cognitive function, unilateral neglect syndrome and most importantly the development of PSD. Depression occurs in roughly one-third of stroke survivors at any one time [10]. PSD is associated with higher mortality and poor functional outcomes [20]. In a comprehensive review performed by the American Heart Association/American Stroke Association (AHA/ASA), it was stressed that several gaps in knowledge of the epidemiology, pathophysiology, outcomes, management and prevention of PSD exist [26]. The consensus statement confirmed the need to further elucidate pathophysiology of PSD as well as the need to further explore the biological factors such as genetic susceptibility, inflammation, alterations in neurotrophic factors, disruption of neural network and alterations in neurotransmitters and psychosocial factors as a matter of priority. This work aims at addressing the biological underpinning behind depression, stroke and inflammation.

### 1.2. Poor Neurological Outcomes in Patients with Post-Stroke Depression

Research has shown that patients with PSD are more likely to be dependent in terms of activities of daily living compared to their non-depressed counterparts [12]. This is confirmed in a meta-analysis of longitudinal studies performed by Blochl et al., which concluded that PSD is linked to poor functional outcomes despite rehabilitation [13]. As a result, there is marked compromise in their quality of life, most especially in domains related to cognitive, emotional, economic and social functioning [15,18]. It is in this regard that van de Weg and colleagues highlight the importance of early recognition of depressive symptoms among stroke patients and initiation of antidepressant therapy if necessary for optimization of rehabilitation therapy [19].

In addition to its negative impact on the quality of life and functional recovery, PSD is also associated with an increased risk of stroke recurrence and mortality [20,21,22,27]. A prospective study including patients with concomitant stroke and depression revealed that PSD doubled the risk for stroke recurrence at the end of one year and resulted in a shorter time period for its occurrence [20]. A similar conclusion was drawn in a meta-analysis of six studies performed by Wu et al. and further adds that ischemic stroke, in particular, augmented the risk for re-stroke [21]. On the other hand, the association of PSD is also related to increased risk of death from all causes. Various studies concluded that depression diagnosed after a neurovascular event increases the risk of mortality at 15 months up to five years after the acute event [22,23,27]. Furthermore, when compared to patients who are depressed and without a history of stroke, the risk of mortality is significantly higher among patients with PSD [24]. This is corroborated by a meta-analysis of observational studies including more than 17,000 patients that concludes PSD has a significant impact on short-term mortality [25,28].

Various mechanisms have been proposed linking PSD and poor neurologic outcomes. Inflammation from stroke and depression has been shown to impact neuroplasticity, as evidenced by the decreased availability of brain-derived neurotrophic factor (BDNF) in synapses [29]. A disproportionate increase in stress also translates to physiological changes predisposing patients to hypertension and cardiac dysrhythmias [30]. Another proposed mechanism is the social signal transduction theory of depression, which highlights the activation of various components of the immune and inflammatory pathways triggered by every individual’s emotional and social experiences [31,32]. Core to this is the role of cytokines, which has also been identified as a mediator in chronic inflammatory processes linked to stroke-related complications [33].

### 1.3. Depression and Inflammation

Inflammation is closely associated with depression [34]. Previous meta-analysis noted an increase in proinflammatory cytokines (TNFα and IL-6) in people with depression [35]. Another meta-analysis of 82 studies found a greater range of changes in cytokines and higher levels of TNFα, IL-6, IL-13, IL-18, IL-12, IL-1RA and sTNFR2 along with a reduced level of pro inflammatory cytokine IFNδ [36]. Eyre et al. noted a wide variety of chemokine levels to be affected with raised levels of CCL2 (MCP-1), CXCL4 and CXCL7, while CCL4 levels were significantly lower in the blood [37,38]. A number of studies reported people with depression to have higher levels of circulating proinflammatory cytokines, interleukin-1β (IL1β) and TNFα and interleukin-6 (IL-6) [39,40,41,42,43,44].

Evidence suggests that antidepressant therapies may reduce depressive symptoms through mediating immune markers with mood effects. Serotonin reuptake inhibitor (SSRI) and serotonin norepinephrine reuptake inhibitor (SNRI) administration was associated with reduced serum levels of TNFα and increased levels of IL-10 [45,46]. In vitro studies using animal macrophages revealed reduction in IL-6 and elevation in IL-10 follows treatment with tricyclic anti-depressant (TCA) drugs such as amitriptyline, and SSRIs such as fluoxetine, suggesting that such effects may be mediated through the inhibition of the nuclear factor kappa light chain enhancer of activated B cells (NF-kB) system [47].

Studies have illustrated the changes in cytokines with antidepressants in humans with lowered levels of IL-1β, IL-4, IL-6 and IL-10 with no definite agreement on specific antidepressant class [48]. Antidepressant-induced immunomodulatory effects were reported with SNRI (venlafaxine) with greater anti-inflammatory effect over SSRI (paroxetine) [49].

### 1.4. Ischaemic Cascade and Inflammation

Ischaemic cascade is a complex event with a series of interconnected cellular and molecular mechanisms with cell death by programmed cell death, swelling or necrosis. Under normal physiological conditions, brain tissue requires a blood flow of 50mL/100g/min to sustain the supply of nutrients and oxygen [50,51]. When an AIS occur, if the cerebral blood flow reduces to less than 10 mL/100 g/min, an ischaemic core will be developed [51,52]. When the blood supply is cut off, a series of complex neurochemical events evolve in time and space. These events are characterised by focal cerebral hypoperfusion, bioenergetic failure, excitotoxicity, acidotoxicity, oxidative stress, microvascular injury, post-ischemic inflammation, blood brain barrier disruption and finally death of neurons, endothelial cells, and neuroglia [53,54].

Microglial cells are thought to be the prime players of the CNS in its own immune and inflammatory responses, while pro-apoptotic pathways are activated as a result of the inflammation [55]. While these processes are usually localized to the CNS, a systemic immune response is also manifested as evidenced by peripheral inflammatory biomarkers such as the neutrophil-to-lymphocyte ratio (NLR) [56,57,58,59,60,61]. Using various depression scales such as HAM-D and Quick Inventory for Depression Symptomatology (QIDS), it has been demonstrated that a high NLR ratio on admission predicted PSD after one month [59,62]. Furthermore, Hu et al. reported in a retrospective study of more than 300 patients that, in conjunction with the platelet-to-lymphocyte ratio, admission NLR is associated with depression at six months after stroke [56].

An important mechanism which leads to the occurrence of depression is low-grade inflammation, which may have an impact in dopaminergic systems [63,64]. A study by Tang et al. illustrated that among patients with ischemic stroke, high-sensitivity CRP (hsCRP) obtained during admission has been shown to correlate with depression at six months post stroke [65]. In a similar manner, homocysteine independently predicted the risk of PSD with an OR of 1.07 (95% CI 1.01–1.22; *p* = 0.013) and that a level of more than ≥ 16.5 mmol/L was linked to a much higher risk of PSD (adjusted OR 6.13, 95% CI 3.32–14.16; *p* < 0.001) three months after the onset of stroke [66]. There is also available evidence that combining both parameters resulted in a greater predictive value in PSD [65,67,68].

Acute phase reactants such as ferritin and leptin have also been shown to have correlation with the occurrence of PSD. Zhu et al. highlights that high serum ferritin obtained during admission correlates with PSD, with levels more than ≥130.15 µg/L associated with an odds ratio of 5.388 (95%CI: 1.725–16.829; *p* = 0.004) [69]. This trend, however, was not replicated in depressed patients without a history of stroke [70,71]. Whether ferritin is implicated in the neuroinflammatory cascade of PSD remains debatable; however, a possible mechanism in which this could be attributed to is that elevated iron levels, as reflected in the elevated ferritin level, is a marker of oxidative stress, which is central to the occurrence of major depressive disorder [72]. Leptin is another acute phase reactant, which has spun interest in the research on PSD. It is a hormone that acts on the receptors in the hypothalamus to signify energy homeostasis [73,74,75]. Among patients who suffered from PSD, there is a trend that leptin levels were positively correlated with the incidence of PSD after one month [75] and at three months [73]. A retrospective study by Lee et al. involving more than 100 patients also provided evidence that elevated serum leptin was associated with depression with an odds ratio of 1.21 (95% confidence interval, 1.01–1.45; *p* = 0.021) [74].

Neopterin is another biomarker that has been shown, in a prospective cohort study, to correlate with a high score using the Hamilton Depression scale [76]. A marker of activation of T-cell and cell-mediated immunity, Neopterin has been shown to be a predictor of PSD among Chinese patients after six months from stroke onset with OR of 1.952 (95% CI, 1.358–2.805, *p* < 0.0001) [77]. Serum BDNF has also been extensively studied as a predictor of mood disorders after cerebrovascular events. Yang et al. describes that low serum BDNF on admission correlates with the occurrence of PSD as early as 14 days post stroke [78]. The same trend was observed in a prospective study at three to six months from the neurovascular event and was further supported by a meta-analysis of four studies including 171 patients with PSD [79,80,81]. The utility of BDNF has been further strengthened with its use as a marker of determining response after antidepressant therapy. Liang and colleagues looked at this laboratory parameter pre- and post-treatment with Venlafaxine and Gingko, and it was concluded that serum BDNF predicted therapeutic response [82]. Lastly, morning serum cortisol, a chemical mediator of the hypothalamic–pituitary–adrenal (HPA) axis, which is central to the pathology of PSD, is also shown to be elevated in patients compared to non-PSD and healthy controls [83] Clearly, these inflammatory biomarkers further provide a backbone to the neuroinflammatory model of post-stroke depression.

Neurochemicals that have protective effects post-stroke have also been proven to have utility as a biomarker in PSD. Adiponectin, which is known for its anti-inflammatory and anti-atherogenic properties, shows inverse correlation with PSD after three months of stroke diagnosis [84]. Similarly, retinoic acid, a metabolite of vitamin A, also possesses anti-inflammatory properties by ameliorating oxidative stress and thereby improving behaviour in animal studies. Correlating with depression diagnostic scales, evidence proves that the development of PSD three months after stroke is likely associated with lower retinoic acid levels at baseline [85,86]. Vitamin D, another neurotrophic factor that provides neuroprotective function by reducing oxidative stress and the burden of inflammation, has also been shown to be a predictor of PSD [87]. A prospective study involving more than 180 stroke patients has shown that low serum vitamin D levels were associated with a diagnosis of PSD at one month post stroke (odds ratio 8.824, 95% confidence interval 2.011–38.720, *p* = 0.004) [88]. This is further complemented by another study which also suggests its predictive value at six months [89]. While vitamin supplementation has been shown to have a potential in decreasing symptoms in patients with depression [90], its role in the treatment of PSD is still unclear.

The inflammatory cascade of events that are well described in both stroke and depression highlights the significance of proinflammatory cytokines, which act as biochemical mediators of this phenomenon [29,54,91,92,93,94,95,96,97]. In the past decade, research has centred on the cytokine hypothesis of depression after stroke. Yang’s publication in 2010 was the first prospective study to evaluate inflammatory cytokines and PSD [98]. Authors concluded that IL-18 is predictive of PSD in the first 2 weeks after stroke [98]. This was corroborated by literature publishing that TNF-α, IL-1B, IL-6 and IL-18 also independently predicted the occurrence of PSD in the acute to subacute period [99,100], and elevated IL-6, IL-10 and TNF-α correlates with PSD at one to three months post stroke [91,98,101,102,103,104,105,106,107,108]. Interestingly, patients who were also diagnosed with PSD at 6 to 12 months still had elevated inflammatory markers, most notably IL6 and IL-18 [98,101,109]. Table 1 summarizes the utility of various cytokines in PSD. Indeed, the presence of these pro-inflammatory cytokines provides a framework that alters the neural milieu to affect neurohormonal metabolism and function.

### 1.5. Neuroanatomical Correlates of PSD

Factors which contribute to the occurrence of PSD are multifactorial, with inflammation, neurohormonal mechanisms and anatomical location thought to be key contributors [12].

Based on the reviewed studies, neuroanatomical correlates of PSD can be categorized into lesion volume, lateralization of lesion, as well as specific lesion locations. Other than the identification of specific site or structure, this review will also discuss additional variables, such as lesion proximity to the frontal pole and the association between subcortical or cortical lesions with PSD.

### 1.6. Lesion Volume

Fifteen studies that discussed the relationship between lesion volume and PSD were identified. Four concluded no significant association [109,115,116,117,118], whilst seven suggested larger volumes to be related to PSD [119,120,121,122,123,124]. Interestingly, two studies showed that larger lesion volumes are only associated with PSD three months and beyond, and not in an acute setting [119,121,122,123,125]. These associations between incidence of PSD and lesion volume might be based on the postulated direct relationship between lesion volume and depression severity [126].

Douven at al reported that PSD in the post- acute phase was associated with frontal lobe lesions with the odds ratio of 1.72, 95% CI 1.34–2.19 and basal ganglia lesions with the odds ratio of 2.25,95% CI 1.33–3.84 [127]. Shi et al. noted that decreased grey matter volume was observed in the prefrontal cortex, motor cortex and limbic system in a cohort of patients with PSD [128]. A functional MRI study explored the involvement of functional networks in three different lesion locations confirming the mechanisms of integrity, locality, compensatory mechanisms as the important factors in emotional networks [128]

It has been postulated that greater lesion volume can be correlated with greater resulting disability post-stroke, which can be associated with the incidence and/or severity of depressive symptoms [129,130]. However, the relationship between lesion volume and depression can also be explained by the greater release of pro-inflammatory cytokines in both strokes and depression [99,131]. It has been established that both PSD and stroke involve an increase in pro-inflammatory cytokines, in particular IL-1β, IL-6, IFN-γ and TNF-α [99]. This was evident from a study by Spalletta, which demonstrated that depression severity, especially in an acute setting, could be correlated to an increase in the levels of IL-6 [100]. Moreover, particular cytokines have also been shown to be highly depressogenic, with one example being the IFN-γ [131], especially based on its influence on serotonin metabolism, the hypothalamus–pituitary–adrenal (HPA) axis as well as its positive feedback on the inflammatory state.

First, IFN-γ may alter serotonin metabolism based on its ability to stimulate the activity of indoleamine 2,3-dioxygenase (IDO), which can degrade tryptophan, a biological precursor of serotonin, into toxic metabolites [132]. Secondly, IFN-γ can influence the HPA axis causing continuous stimulation of adrenocorticotrophic hormone (ACTH) and cortisol. A study has shown that administration of IFN-γ increases the levels of both hormones, which subsequently lead to the occurrence of depressive symptoms [133]. The elevated level of glucocorticoids can also affect mitochondria, causing respiratory chain dysfunction and increase in reactive oxygen species, which may further perpetuate neuronal injury and cellular death from the original lesion [134,135]. Third, IFN-γ is a potent inducer of other pro-inflammatory cytokines, such as IL-6, IL-1β and TNF-α, which may further the inflammatory response following the original stroke insult [131].

### 1.7. Lesion Laterality

With regards to lateralization of PSD, 14 out of 25 studies showed no association between the hemispheric lesion locations and PSD, whilst 6 reported significant association with left-sided lesions [22,27,115,118,136,137,138,139,140,141,142,143,144,145,146,147,148,149,150,151]. The inconsistent outcomes from the studies might be due to the currently poor understanding of the different hemispheric roles in moods and behaviour. Older theories simplified the roles of the right hemisphere as having a negative perception of the world and the left with a positive view [152]. This theory was later developed to include the roles of dominance and contralateral release, stating the possibility that lesions in the dominant hemisphere might dampen the inhibitory role against the non-dominant hemisphere, which manifests as depression [153,154].

Of the 40 studies that reviewed the possible relationship between lesion location(s) and PSD, as presented in Table 2, nearly one-third (*N* = 15) reported no significant association. However, amongst those that did, PSD appeared to be associated more with lesions located in the anterior part of the brain (*N* = 14), which included the frontal lobe (*N* = 10), anterior cerebral artery territory (*N* = 3) and prefrontal cortex *N*= 1). In addition, two studies also highlighted the significant correlation between PSD and the proximity of the lesion to the frontal pole [137,148].

Other locations reported to be involved in PSD are those within the subcortical structures of the brain (*N* = 13). More specifically, these include the basal ganglia (*N* = 8), internal capsule (*N* = 4), and corona radiata (*N* = 1). Interestingly, despite the lacking support for hemispheric association with PSD, as discussed previously, more than one-third of the studies demonstrating associations between frontal (*N* = 5) and subcortical lesions (*N* = 5) with PSD highlighted statistical significance between PSD and lesions in the left hemisphere.

With these relative inconsistencies in the neuroanatomical localization of PSD, improved understanding on specific lesion location or neurocircuitries associated with PSD is essential. This can be based on current knowledge of known areas affected in PSD, the vascular depression hypothesis, as well as previous lesion localization studies for PSD.

The theories relating to PSD have been based on the monoamine theory of depression, which stipulates that downregulation of both pre- and post-synaptic receptors associated with serotonin, noradrenaline and dopamine neurotransmitters may explain for the manifestation of depressive symptoms [170,171] Neuroimaging studies have also shown associations between PSD and reduced volumes in the caudate, putamen and frontal cortex [172].

In 1977, Robinson and Bloom built on this hypothesis by delineating significant associations between reduced mood and reduced serotonin and noradrenaline levels in the limbic structures, arguably due to interruptions of ascending neurons from brainstem nuclei to the cerebral cortex, particularly to the prefrontal cortex. Another study also highlighted disruptions in the dopaminergic pathway, affecting the mesolimbic reward circuitry, leading to the symptom of anhedonia.

One of the more targeted studies for neuroanatomical localization of PSD was conducted by Mayberg [172], which supported the likelihood for the involvement of limbic structures, reward circuitry and anterior temporal cortex, with more specific regions being the hypothalamus, hippocampus, amygdala, insula and the cingulate cortex [172]. This hypothesis is further supported by work showing that structures such as the anterior cingulate cortex (ACC), amygdala and anterior insula are involved in mood and emotional regulation [172]. Additionally, the presence of WMH in the frontal cortex and basal ganglia have also been postulated to be associated with depressive symptoms [173] These were evident in light of (positron emission tomography)PET studies by Mayberg [172,173,174] indicating the association between sadness with changes in regional cerebral blood flow in the cingulate cortex and insula, as well as the correlation between glucose uptake as a proxy of cingulate metabolism in predicting depressive symptom remission [172,174]. However, in contrast to earlier findings, more recent studies have shown that PSD indeed involves a more diverse area implicating both cortical and subcortical structures [175,176].

In addition, functional studies in the likes of PET and single photon emission computed tomography (SPECT)have shown that reduced metabolism in the dorsolateral prefrontal cortex, medial prefrontal cortex, basal ganglia and the cingulate cortex was associated with a paradoxical increased activity in the amygdala [177]. The latter is seemingly consistent with the finding of more pronounced amygdala activation in the presence of 5-HTTLPR s/s genotype, which along with the STin2 VNTR polymorphisms of the serotonin transporter gene (SERT) have been known to increased predisposition to psychiatric comorbidities, including PSD [178,179,180]. More novel studies, such as ones using Voxel-based analysis, have also supported the role of ACC and the dorsomedial prefrontal cortex in PSD, as critical components of the frontal-subcortical circuitry (FCC) [181]. Further to this, Bora (2012) also found a significant association between decreased grey matter volume in the ACC and in the dorsomedial prefrontal cortex with patients presenting with multiple episodes [179]. In contrast, firstly presenting cases corresponds more with reduced volumes in the amygdala and parahippocampal area. In addition, individuals diagnosed only with PSD without other psychiatric comorbidities exhibited reduced activity in the right pre-central or dorsolateral frontal grey matter [63,180]. Accordingly, loss of grey matter volumes in these areas may be a result of lower glial density and neuronal cell reductions [182], and likely perpetuated by the cascade of pro-inflammatory processes, neuronal insults and reduced neuroplasticity. This was consistently demonstrated by a PET study highlighting the role of IFN-γ in dampening the metabolic activities in structures such as the basal ganglia and dorsal ACC [164,183].

Another concept that may contribute to improved understanding of lesion localization of PSD is the vascular depression hypothesis, as initially drawn by Alexopoulos in 1997 [182,183]. Vascular depression is considered as one subtype of late-life depression, occurring in the elderly aged 65 and beyond. This hypothesis can be defined both clinically, in accordance to the age of onset and presence of vascular risk factors, as well as on the basis of neuroimaging [184,185]. Looking at the latter, many consider vascular depression as a small-vessel disease, characterized by the presence of white matter hyperintensities (WMH), lacunar infarcts and cerebral microbleeds [182,183,184,185]. The mechanism whereby these small vessel pathologies contribute to vascular depression can be further distinguished based on the degree of vascular burden and strategic lesion locations [186]. Not many studies have concluded the associations between lacunar infarcts and cerebral microbleeds with depression, but a few have shown significant associations between WMH and lesion volumes, as directly correlated with the severity of depressive symptoms [186].

With respect to the specific areas for depression, Krishnan concluded the involvement of the striato-pallido-thalamo-cortical pathway [187]. Meanwhile, studies have also demonstrated the involvements of the dorsolateral prefrontal cortex (DLPFC) [186,188]. This is in agreement with established theories of PSD with presence of neuroinflammatory state and the demonstrated treatment efficacy of transcranial magnetic stimulation targeted to the DLPFC [186,188].

As Robinson considered PSD to be a subset of vascular depression [188], few similarities between PSD and vascular depression can be appreciated. One of the similarities is the involvement of subcortical structures in their pathologies [189,190]. This was evident through this review, with PSD being associated with subcortical lesions in four out of six studies, with the other two showing no significant association [101,121,147,150,168,191]. Few studies also showed statistically significant associations between markers of vascular burdens, such as presence of multiple infarcts and cerebral microbleeds, with incidence of PSD in three months [168,178]. Some of the clinical features of the two are also identical with greater prevalence of apathy and social isolations, in conjunction with both of their relationships with cognitive impairment and interruptions in activities of daily living [189]. However, unlike PSD, the concept of vascular depression to date is still leaning towards neuroimaging-based diagnosis instead of clinical. Additionally, some studies concluded that vascular depression is more of a consequence of small-vessel pathologies, whilst PSD affects larger vessels [173,186]

An MRI-based study by Tang in 2011 concluded that the presence of infarct in the frontal subcortical circuit (FSC) bears statistically significant association with the occurrence of PSD [160]. FSC consists of five circuits with three of them being DLPFC, the anterior cingulate circuit and the orbitofrontal circuit [192]. These are critical circuitries for executive functioning, motivation and emotional regulations [192,193]. In this review, despite 14 studies showing no significant association between PSD and lesion location, most of the studies that do provided some support for the involvement of FSC in PSD. Neurons in the caudate nucleus and pallidum, for example, have also been known to be part of both the dorsolateral prefrontal circuit and anterior cingulate circuit [194]. Moreover, these are also two areas long-known to be involved in the pathogenesis of PSD. Aside from the connection within the basal ganglia [171,195,196] Singh has shown involvement of the lateral division of the orbitofrontal circuit, as one responsible for emotional regulation [137,168]. This is also evident through the significant correlation between inferior frontal lesion and PSD [194].

Other than its purpose as potential predictor for PSD, lesion location can also be used to explain the severity of depressive symptoms. Despite lacking adequate evidence, one example of this is the proximity of the anterior border of the lesion to the frontal pole, which was shown by Sinyor to be related to the severity of PSD [146].

Robinson and colleagues were the first to correlate anatomy and behavioural manifestation in animal models [193]. Subsequently, various studies have linked PSD with brain laterality, specific neuroanatomical location and stroke subtype. A cross-sectional study involving 28 patients with PSD concluded that left hemispheric cortical and subcortical infarcts were associated with the development of the former [146]. This was further alluded by another study which proved that left hemispheric lesions, particularly in the frontal lobe, were independently associated with severe depression [194]. A systematic review involving more than 5500 patients refuted this claim, which was further corroborated by another meta-analysis two years later [127]. However, there is evidence to suggest that left-sided lesions are usually associated with PSD in the acute phase [194], while right hemispheric lesions resulted in the later during the subacute phase [127].

State-of-the-art technology that has emerged over the years has allowed researchers to correlate neuroanatomy and functional changes with respect to mood-related conditions post stroke. Shi et al. performed voxel-based morphometry and functional magnetic resonance imaging (fMRI) in 30 patients with PSD [128,197]. In their analysis, it has been demonstrated that decreased grey matter, particularly at the prefrontal cortex, limbic system and the motor cortex, were the main culprits to PSD [128,197]. This was further supported by another study which showed that disruption of the functional connectivity of the insular cortex, left putamen and right superior longitudinal fasciculus correlated with worse Hamilton Depression (HAM-D) Rating Scale score for depression [198]. Known collectively as the limbic-cortical-striatal-pallidal-thalamic (LCSPT) circuit, it has been demonstrated that patients with post-stroke depression have distinct white matter microstructural changes as typified by fractional anisotropy (FA) and mean kurtosis (MK) levels [198,199]. In particular, Shen and colleagues have provided evidence that FA of the left frontal lobe and MK levels of bilateral frontal lobes were substantially smaller when compared to their non-PSD counterparts [198]. The same trend was observed in the bilateral anterior limbs of the internal capsule; however, Yasuno showed a negative correlation between depressive symptoms and FA values 6 months after acute ischemia [199].

### 1.8. Inflammation-Related Genetic Polymorphisms Associated with PSD

There is compelling evidence that genetic mechanisms lead to the activation of various cell lineages and inflammatory factors that play a key role in the pathology of PSD. It has been substantiated in the literature that serotonin is core to the pathogenesis of PSD. In particular, the role of the serotonin transporter gene and the tryptophan hydroxylase 2 (TPH2) gene, both of which are essential in serotonin synthesis, have been evaluated in human and animal studies to correlate with PSD [200,201]. In a study involving 199 Chinese patients with PSD, it has been demonstrated that the serotonin transporter-linked polymorphic region (5-HTTLPR) polymorphism was linked to patients’ susceptibility to PSD [200]. Moreover, a case control study comparing stroke survivors with and without depression has also provided evidence that the presence of mutations in the 5-HTTLPR gene resulted in a three-fold odds of acquiring PSD [201,202]. The significance of this genetic mutation was also replicated in a meta-analysis of seven trials, which has shown that homozygosity to 5-HTTLPR (5-HTTLPR) polymorphism was significantly associated with PSD, while the heterozygous and the recessive models were shown to be protective [177]. On the other hand, genetic abnormalities involving the TPH2 have also been evaluated in PSD. Genotypic studies of more than 300 Korean patients have shown that the presence of *TPH2* rs4641528 C was predominant among these patients and suggests that homozygosity to this allele makes patients susceptible to PSD [203]. This finding is further strengthened in a study published by Tsai et al., which showed that patients who responded to selective serotonin reuptake inhibitors had a higher proportion of heterozygous carriers of this gene compared to non-responders [204]. In addition to TPH2, there is also evidence that methylation of the brain-derived neurotrophic factor (BDNF) gene, which is key in neuronal maturation and synaptic plasticity, increases susceptibility to PSD and predicts response to treatment [205,206]. A study by Kim and colleagues looked into the relationship of BDNF methylation status and PSD and has shown that increased burden of the former is associated with higher incidence of PSD and worse symptoms [207]. Similarly, animal models have also shown that BDNF expression in the hippocampus and cerebellum of rats was significantly lower in patients with depression post stroke compared to normal controls [79,208,209]. This genetic biomarker has also proven its utility in assessing a patient’s response to treatment. Fluoxetine, an SSRI which is used in the treatment of PSD, has been shown to upregulate the expression of BDNF synthesis in mice hippocampus, which improved depressive signs [210].

Another identified receptor that has been shown to play an important role in PSD is the expression of the P2X4 (P2X4Rs) [211]. These receptors, which are ubiquitous in CNS cell lineages, particularly in microglia and monocytes, have been shown to reduce the burden of depression and improve stroke recovery in mice models [211]. Animal studies have provided evidence that myeloid-specific (MS) P2X4R knock-out (KO) mice exhibited depressive phenotype regardless of the size of stroke [211]. This is likely explained by the alteration in the concentration of tyrosine hydroxylase and dopamine receptors pre and post-synaptically, which is central to the pathogenesis of depression [212]. These receptor alterations have also resulted in depression-related behaviours such as increased ethanol intake in transgenic mouse models [213]. On the other hand, activation of P2X2 receptors in the medial prefrontal cortex has been shown to have antidepressant effects [214]. Mouse models have provided evidence that modulation of P2X2 receptors by inducing ATP release from astrocytes has resulted in the alleviation of depression [214]. While the clinical utility of P2XR modulation has been explored in neuropsychiatric conditions such as alcohol addiction [213], its role in PSD has not been fully elucidated. Another genetic biomarker that has been looked into is apolipoprotein E (APOE) expression, which is another genetic biomarker for neurodegenerative conditions. It is an apoprotein that is known to regulate lipid homeostasis and also plays a critical role in neuronal repair [215]. A study of Chinese patients with PSD has shown that the presence of APOE polymorphism is linked to an increased risk of PSD with an odds ratio of 3.17 for the rs429358-TC allele and 11.24 for the rs429358-CC allele [216].

## 2. Anti-Inflammatory Treatment in PSD

There is substantial evidence to suggest agents that target inflammation may have a putative role in the treatment of PSD. Both human and animal studies show amelioration of depressive symptoms using various regimens among post-stroke patients.

### 2.1. Anti-Inflammatory Properties of Herbal Medications for PSD

Herbal medications have been extensively investigated for PSD in various populations. An experimental study on the herbal medication paeoniflorin improved depressive -like behaviour among rat models, similar to the effects of fluoxetine [217]. A mechanism proposed for its benefit is the increased BDNF and *p*-CREB expression in the hippocampus [217]. Another animal study confirms the utility of Yi-nao-jie-yu prescription for PSD in rat models [209]. Its potential beneficial effects is attributed to the upregulation of Notch signalling genes, which is key to its neurogenesis [209]. Studies in human subjects have also provided substantial proof on the utility of traditional herbal medicines for PSD. A meta-analysis of randomized clinical trials demonstrates that Chai Hu Shu Gan San, a Chinese herbal medication composed of three subcomponents with anti-inflammatory and antioxidant properties, has benefits for post-stroke depression [218]. The Korean traditional medicine Sihogayonggolmoryeo-tang has also shown to have antidepressant effects in a meta-analysis performed by Kwon and colleagues [219]. The anti-inflammatory effect of one of its components, *Bupleuri Radix,* is attributed to the increased levels of nerve growth factor and brain-derived neurotrophic factor [220]. Curcumin is another traditional medication, which exerts its antidepressant effects among stroke patients by inhibiting P2X7R and subsequently deactivating calcium-mediated inflammatory effects related to PSD [221] While the beneficial effects of traditional medicine may be convincing, a systematic review of randomized control trials comparing various Chinese herbal medicines and fluoxetine shows otherwise [222]. This study concludes that most of the clinical trials had a high risk of bias and therefore draws no firm conclusion to ascertain safety and efficacy [218,219,222]. To date, there are no existing guidelines that recommend herbal medications for poststroke depression.

### 2.2. Anti-Inflammatory Properties of Antidepressants

Various antidepressants are known for their pleiotropic properties including their anti-inflammatory mechanism. As shown in post-ischemic rat models, it has been demonstrated that fluoxetine exhibits a dose-dependent reduction in the activation of cellular inflammatory mediators such as microglia and neutrophils and suppresses the activity of NF-kappaB [223]. Fluoxetine also reduced the levels of inflammatory cytokines such as TNF-α, IL-1β and IL-6 in experimental studies [224]. Furthermore, fluoxetine injections in rat models enhanced neurogenesis and prevented a pathological increase in stem cell recruitment in the hippocampus [224]. Similarly, citalopram demonstrates neuroprotective effects by decreasing oxidative stress, inflammation and apoptosis [225]. Additionally, the natural antidepressant hyperforin also improves post-stroke depression and post-stroke isolation in rat models by inhibiting TGF-β, resulting in the promotion of hippocampal neurogenesis [226,227]. While there is a growing number of animal studies looking into the anti-inflammatory effects of various antidepressants, these benefits have not been established in human studies to date.

### 2.3. Antioxidants and Other Anti-Inflammatory Medications for PSD

The production of reactive oxygen species as a result of oxidative stress and lipid, protein and DNA damage has also shown to be key in the pathophysiology of PSD [228,229]. It is in this regard that antioxidants such as polyphenols might play a key role as a therapeutic agent [229]. The antioxidant components of green tea such as polyphenol, theanine, glutamine and caffeine have also been shown to be beneficial for PSD [230]. Minocycline, on the other hand, has also been studied for the treatment of depression in mice with global cerebral ischemia [231]. This is likely secondary to the upregulation of neuroprotective cytokines and the reduction in hippocampal degeneration [231,232]. These observations and theoretical implications have yet to be replicated in human studies.

## 3. Conclusions

Depression is not an uncommon post-stroke complication, and it impacts every patient’s functional recovery, quality of life and predisposes to a higher risk of stroke recurrence and mortality. One of the key factors contributing to the occurrence of post-stroke depression is the heightened state inflammation, which affects neural and systemic pathways as evidenced by various biomarkers. Furthermore, neuroanatomical correlates primarily involving the frontal cortical and subcortical structures also play a critical role for this post-stroke complication. Various studies support the potential role of therapy aimed at decreasing inflammation in post-stroke depression, but clinical trials are limited to validate its use. More studies are necessary to look at treatment strategies that potentially target the neuroinflammatory basis of this growing public health concern.

## Figures and Tables

**Table 1:** Inflammatory biomarkers in post-stroke depression.

Author, Year	Design, Sample Size	Biomarker	Biomarker Extraction (With Respect to Stroke)	PSD Scale Used	PSD Assessment (With Respect to Stroke)	Findings
Yang et al., 2010 [98]	Prospective *N* = 100	IL-6, IL-18 and TNF- α	D1 and D7	HAM-D, MADRS	6 months	Serum IL-18 on day 7 after admission may predict the risk of post-stroke depression both at the acute stage of stroke and at 6 months post-stroke.
Su et al., 2012 [103]	Prospective *N* = 104	IL-6/IL-10 and TNF-α/IL-10	1st, 3rd, 6th, 9th and 12th month after stroke.	HAM-D	1st, 3rd, 6th, 9th and 12th month after stroke.	There were significant increases in the cytokine’s interleukin-6 (IL-6), interleukin-10 (IL-10), tumour necrosis factor α (TNF-α) and interferon-γ, and the ratios of IL-6/IL-10 and TNF-α/IL-10 were also elevated.
Spalletta et al., 2013 [100]	Prospective *N* = 48	IL-6	D3	HAM-D	D3, 6, 14	Increased IL−6 plays a key role in the onset of depressive disorders, apathy/amotivation, somatic symptoms of depression, and neurological/functional symptoms, resulting in higher disability and poor outcome of stroke patients.
Kang et al., 2016 [101]	Prospective *N* = 286	IL-6 and IL-18	1 week	DSM IV, HAM-D	2 weeks	Higher IL-6 and IL-18 levels were independently associated with depressive disorders within 2 weeks and at 1 year after stroke.
Jiao et al., 2016 [105]	Prospective *N* = 355	CRP, IL-1β, IL-2, IL-6 and TNF-α	D2	BDI	12 months	The risk of PSD elevated with increased interleukin (IL)-6 expression levels [hazard ratio (HR) = 3.18; 95% confidence interval (CI), 1.37–7.36].
Kim et al., 2017 [102]	Prospective *N* = 286	TNF-α, IL-1B	2 weeks	DSM IV	2 weeks and 1 year	Higher TNF-α levels were associated with PSD at 2 weeks in the presence of the -850T allele with a significant interaction term; higher IL-1β levels were associated with PSD at 2 weeks in the presence of the -511T allele with a borderline significant interaction term and with any +3953C/T polymorphism without a significant interaction term.
Li et al., 2017 [110]	Prospective *N* = 280	hSCRP, TNF-α, IL-6	-	HAM D	3 months	TNF-α, IL-6 and Barthel index are the independent risk factors of PSD in acute phase, so do NIHSS score and Barthel index in recovery period.
Meng et al., 2017 [111]	Prospective *N* = 83	TNF-α	D1	HAM-D	1 week	High HAMD scores (OR: 2.38, 95% CI: 1.61–3.50, *p* < 0.001) were independent risk predictors for PSD and so were lower dopamine levels (OR: 0.64, 95% CI: 0.45–0.91, *p* = 0.014), lower 5-hydroxytryptamine levels (OR: 0.99, 95% CI: 0.98–1.00, *p* = 0.046), higher tumour necrosis factor-α levels (OR: 1.05, 95% CI: 1.00–1.09, *p* = 0.044), and lower nerve growth factor levels (OR: 0.06, 95% CI: 0.01–0.67, *p* = 0.022).
Wang et al., 2018 [108]	Prospective *N* = 152	IL-6, hsCRP, vitamin D	D0	HAM-D	1 month	Serum levels of vitamin D and interleukin-6 were associated with the development of PSD after adjusted possible variables (OR = 0.976, 95% CI: 0.958-0.994, *p* = 0.009; OR = 1.029, 95% CI: 1.003-1.055, *p* = 0.027).
Xu et al., 2018 [112]	Prospective *N* = 333	MIF, HCY, CRP and (IL-6)	D1	BDI	3 months	In the patients with major depression, plasma levels of MIF were higher compared with those in patients free from depression [27.3(IQR, 23.5-34.9) ng/mL vs. 20.9(IQR, 17.0–24.8) ng/mL; Z = 8.369, *p* < 0.001]. For each 1 unit increase in MIF, the unadjusted and adjusted risk of PSD increased by 18% (odds ratios [OR]: 1.18; 95% confidence interval [CI], 1.13–1.23, *p* < 0.001) and 11% (1.11; 1.02–1.16, *p* = 0.001), respectively.
Kozak et al., 2019 [113]	Cross-sectional	TNF-α, IL-1 β, IL-18, BDNF, and NSE	D0	DSM IV	-	There is no significant relationship between major depression and basal proinflammatory cytokines (TNF-α, IL-1 β, IL-18), BDNF and NSE.
Hu et al., 2019 [114]	Prospective *N* = 376	IL-17 and IL-6	2 weeks	DSM IV HAM-D 17	3 months	IL-17 and IL-6 at 2 weeks after admission are all independent predictors of the occurrence of PSD at 3 months after stroke.
Chen, 2020 [104]	Metanalysis *N* = 889	IL-6	-	DSM IV HAM D	-	The serum concentrations of interleukin-6 (IL-6) and tumour necrosis factor-alpha (TNF-α) were higher in the PSD group, compared with the non-PSD group (IL-6: SMD = 1.26, 95% CI = [0.55, 1.97], *p* < 0.001; TNF-α: SMD = 0.61, 95% CI = [0.13, 1.10], *p* = 0.010).

**Table 2:** Different lesion locations associated with PSD based on the reviewed studies.

Lesion Locations	Studies (*N =* 42)
No significant associations (*N* = 14)	[117,118,119,133,138,142,146,152,153,154,155,156]
Anterior/ACA vascular territory (*N* = 5)	[121,125,127,157,158]
Left anterior (*N* = 1)	[157]
Posterior (*N* = 1)	[109,159]
Proximity to the frontal pole (*N* = 2)	[121,145]
Frontal lobe (*N*= 10)	[137,140,141,155,160,161,162,163,164,165]
Left frontal lobe (*N* = 3)	[141,161,162]
Inferior frontal lobe (*N* = 1)	[163]
Left prefrontal cortex (*N*= 2)	[22,27]
Temporal lobe (*N*= 4)	[141,160,165,166]
Left temporal lobe (*N* = 1)	[141]
Basal ganglia (*N* = 8)	[22,115,147,161,164,165,167,168]
Left basal ganglia (*N* = 3)	[115,141,161]
Caudate (*N* = 2)	[147,168]
Putamen (*N* = 1)	[168]
Pallidum (*N* = 2)	[167,168]
Left posterior pallidum (*N* = 1)	[167]
Lentiform (*N* = 2)	[115,147]
Internal capsule *N* = 4)	[120,147,165,169]
Left internal capsule (*N*= 1)	[123,169]
Anterior limb of internal capsule (*N* = 1)	[147]
Posterior corona radiata (*N* = 1)	[168]
Brainstem (*N* = 1)	[161]
Occipital lobe (*N* = 1)	[123]

## Data Availability

Not applicable.
